# Integrative genomics analysis of nasal intestinal-type adenocarcinomas demonstrates the major role of CACNA1C and paves the way for a simple diagnostic tool in male woodworkers

**DOI:** 10.1186/s13148-021-01122-5

**Published:** 2021-09-25

**Authors:** Patrice Gallet, Abderrahim Oussalah, Celso Pouget, Gunnar Dittmar, Celine Chery, Guillaume Gauchotte, Roger Jankowski, Jean Louis Gueant, Rémi Houlgatte

**Affiliations:** 1 INSERM U1256, NGERE-Nutrition, Genetics, and Environmental Risk Exposure, Faculty of Medicine of Nancy, University of Lorraine, 54000 Nancy, Vandoeuvre-lès-Nancy, France; 2 ENT Department, CHRU NANCY, 54511 Vandoeuvre-lès-Nancy, France; 3 Pathology Department, CHRU NANCY, 54511 Vandoeuvre-lès-Nancy, France; 4 Proteome and Genome Research Unit, Department of Oncology, Luxembourg Institute of Health, Luxembourg, Luxembourg

**Keywords:** Metaplasia, CACNA1C, SLC26A10, CDX2, Wood dust exposure

## Abstract

**Background:**

Nasal intestinal-type adenocarcinomas (ITAC) are strongly related to chronic wood dust exposure: The intestinal phenotype relies on CDX2 overexpression but underlying molecular mechanisms remain unknown. Our objectives were to investigate transcriptomic and methylation differences between healthy non-exposed and tumor olfactory cleft mucosae and to compare transcriptomic profiles between non-exposed, wood dust-exposed and ITAC mucosa cells.

**Methods:**

We conducted a prospective monocentric study (NCT0281823) including 16 woodworkers with ITAC, 16 healthy exposed woodworkers and 13 healthy, non-exposed, controls. We compared tumor samples with healthy non-exposed samples, both in transcriptome and in methylome analyses. We also investigated wood dust-induced transcriptome modifications of exposed (without tumor) male woodworkers’ samples and of contralateral sides of woodworkers with tumors. We conducted in parallel transcriptome and methylome analysis, and then, the transcriptome analysis was focused on the genes highlighted in methylome analysis. We replicated our results on dataset GSE17433.

**Results:**

Several clusters of genes enabled the distinction between healthy and ITAC samples. Transcriptomic and IHC analysis confirmed a constant overexpression of CDX2 in ITAC samples, without any specific DNA methylation profile regarding the CDX2 locus. ITAC woodworkers also exhibited a specific transcriptomic profile in their contralateral (non-tumor) olfactory cleft, different from that of other exposed woodworkers, suggesting that they had a different exposure or a different susceptibility. Two top-loci (CACNA1C/CACNA1C-AS1 and SLC26A10) were identified with a hemimethylated profile, but only CACNA1C appeared to be overexpressed both in transcriptomic analysis and in immunohistochemistry.

**Conclusions:**

Several clusters of genes enable the distinction between healthy mucosa and ITAC samples even in contralateral nasal fossa thus paving the way for a simple diagnostic tool for ITAC in male woodworkers. CACNA1C might be considered as a master gene of ITAC and should be further investigated.

*Trial registration*: NIH ClinicalTrials, NCT0281823, registered May 23^d^ 2016, https://www.clinicaltrials.gov/NCT0281823.

**Supplementary Information:**

The online version contains supplementary material available at 10.1186/s13148-021-01122-5.

## Background

Among sinonasal tumors, intestinal-type adenocarcinomas (ITACs) form a very particular subgroup: These tumors are indeed typically induced by chronic wood dust exposure, which might be considered as a *sine qua non* condition [1, 2]. The risk of developing an ITAC increases with exposure duration (increased after 1 year), exposure intensity (increased above 1 mg dust/m^3^) and latency (after 20 years) [3]. While a short exposure (< 3 years) might be sufficient, there is a very significant increase in risk with duration of exposure (OR = 5.3 for less than 5 years of exposure, 10.7 for 10–19 years and 36.7 for more than 30 years) [4]. ITACs development is usually delayed and average latency (mean time to onset of the disease after the first exposure to wood dust) is long (28–40 years [3, 5], so that 90% of patients are over 50 years of age [1, 2]. A huge majority of woodworkers are male so that ITACs affect men in almost all cases.

It has been demonstrated that ITACs arise from the olfactory cleft, which is the confluence of two tissues of different embryological origin: respiratory epithelium, and olfactory epithelium [6, 7]. For some authors, ITACs might be preceded by a phase of metaplasia [8–13]. The transformation of the normal epithelium into an ITAC relies on a key event, the overexpression of *CDX2* [1, 11]. This phenomenon is constant, as *CDX2* expression seems mandatory for the intestinal organization of these tumors [1]. The other genomic abnormalities observed (such as p53 mutations and loss of heterozygosity) are more inconstant, and associated with more advanced forms of adenocarcinomas, thus suggesting that they could be late events in ITACs’ natural history [1, 2]. The precise role of *CDX2* in carcinogenesis is, however, not precisely established: Chronic inflammation might trigger *CDX2* expression thus leading to metaplasia [1, 2, 11]. However, this has never been confirmed.

Early diagnosis would offer a significant benefit: the 5-year disease-free survival is 100% for T1 tumors, 85–100% for T2, and < 40% for T4 [5]. Unfortunately, the diagnosis is usually delayed because symptoms are not very marked and not specific. Organized screening of exposed woodworkers is therefore likely to enable early diagnosis (metaplasia or small tumors), but screening currently relies only on endoscopy and the olfactory cleft is a narrow space, sometimes difficult to access and explore [14]. Biopsies may be very difficult to perform in consultation given the anatomical conditions (narrowness of the olfactory cleft, septal deviation, polyps, etc.). It could therefore be important to have an alternative screening method. But there is to date no study on transcriptomic and methylation abnormalities in woodworker’s nasal epithelium.

Therefore, we proposed an original approach to investigate wood dust-induced modifications: We developed a noninvasive sampling method to take samples from the olfactory cleft in normal volunteers (non-exposed), exposed woodworkers and exposed woodworkers with a tumor, with the aim to investigate wood dust-induced transcriptomic and methylation modifications. The first objective of this study was to identify the main transcriptomic and methylation modifications in the olfactory cleft mucosa between woodworkers presenting a tumor and healthy controls. The second objective of this study was to identify the main transcriptomic modifications between tumor cells, wood dust-exposed non-tumor mucosa and non-exposed mucosa. Overall, this work might contribute to ITACs carcinogenesis understanding and help in defining a new screening method.

## Results

### Population

**Fig. 1:**
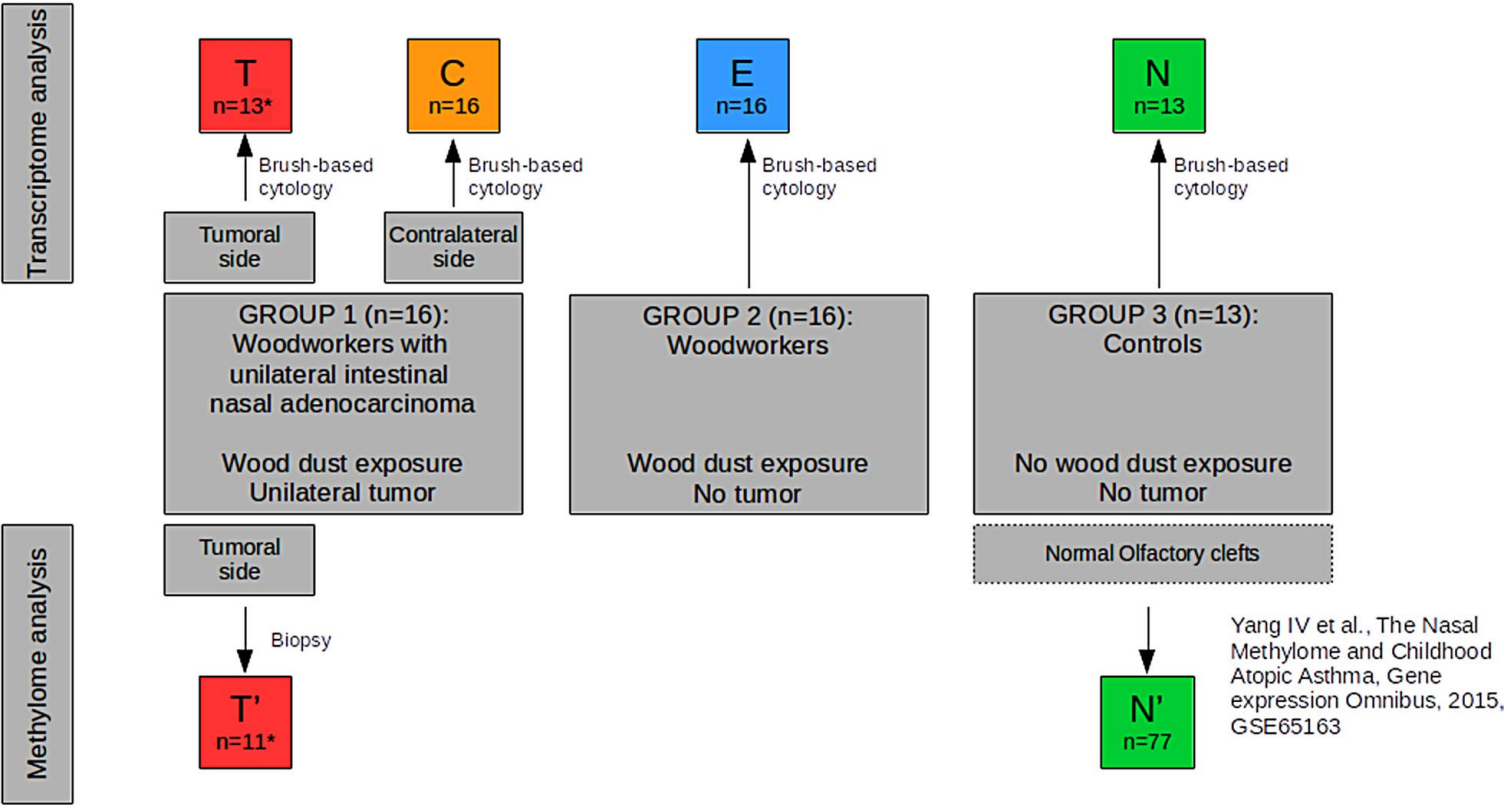
General design. The 4 groups of samples obtained by brush-based cytology (T = tumor, C = Contralateral to tumor, E = wood dust-exposed mucosa without tumor, N = Normal unexposed mucosa) were compared with each other in transcriptome analysis. Tumor biopsies were compared with samples of healthy mucosa (GEO database GSE65163) [17] in methylome analysis

The general design is depicted in Fig. 1 for a better understanding. Sixteen woodworkers with unilateral intestinal-type sinonasal adenocarcinomas (group 1), 16 woodworkers without cancer (group 2) and 13 healthy subjects (group 3) were included. Exposure to wood dust and main other carcinogens is presented in Table 1. Woodworkers with adenocarcinomas were slightly older than controls. The latency was longer in group 1 (adenocarcinoma) than in group 2, while exposure duration was longer in group 2. There was no other difference between groups. In particular, we could not identify any differences between group 1 and 2 in the wood types processed or in woodworkers activities.

**Table 1:** Population description

	Group 1 (*n* = 16)	Group 2 (*n* = 16)	Group 3 (*n* = 13)	Comparison 1 vs 2 (*p* =)	Comparison 1 vs 3 (*p* =)	Comparison 2 vs 3 (*p* =)
Age (years)	69.0 ± 9.5	58.8 ± 6.1	60.4 ± 14.9	0.002	ns	ns
ITAC	Yes	No	No			
Wood dust exposure	Yes	Yes	No			
Exposure duration (years)	32.2 ± 17.9	43.3 ± 6.3		0.0257	-	-
Delay/first exposure (years)	54.5 ± 9.6	36.2 ± 28.5		0.0005	-	-
Other carcinogens						
Tobacco (current use) (*n* =)	2	2	1	ns	ns	ns
Tobacco (current or former use) (*n* =)	12	8	7	ns	ns	ns
Asbestos exposure (*n* =)	2	2	4	ns	ns	ns
Other carcinogen (formaldehyde…) (*n* =)	1	1	1	ns	ns	ns

Woodworkers with adenocarcinomas were slightly older than controls. The latency was longer in group 1 than in group 2, while exposure duration was longer in group 2. There were no other difference between groups in particular in the wood types processed or in woodworkers activities for group 1 and 2

### Brushing technique and samples collection

#### Brushing technique evaluation

Swabbing was relatively easy to perform and well tolerated: There were no side effects and it was usually painless (3 volunteers refused local anesthesia, mean numerical rating scale: 2.7 ± 1.8/10, maximum: 8 in a woodworker with a huge septal deviation). The sampling method was found to have an excellent “acceptability,” and “perceived usefulness” (9.1 ± 1.4/10 and 8.4 ± 1.4/10, respectively) and all volunteers “would accept to do it again if needed.”

#### Samples

In three woodworkers, tumor-side samples (T) were finally not included for transcriptome analysis, because a prior recent but incomplete tumor exeresis might have biased results for this side.

### Global microarray data analysis

**Fig. 2:**
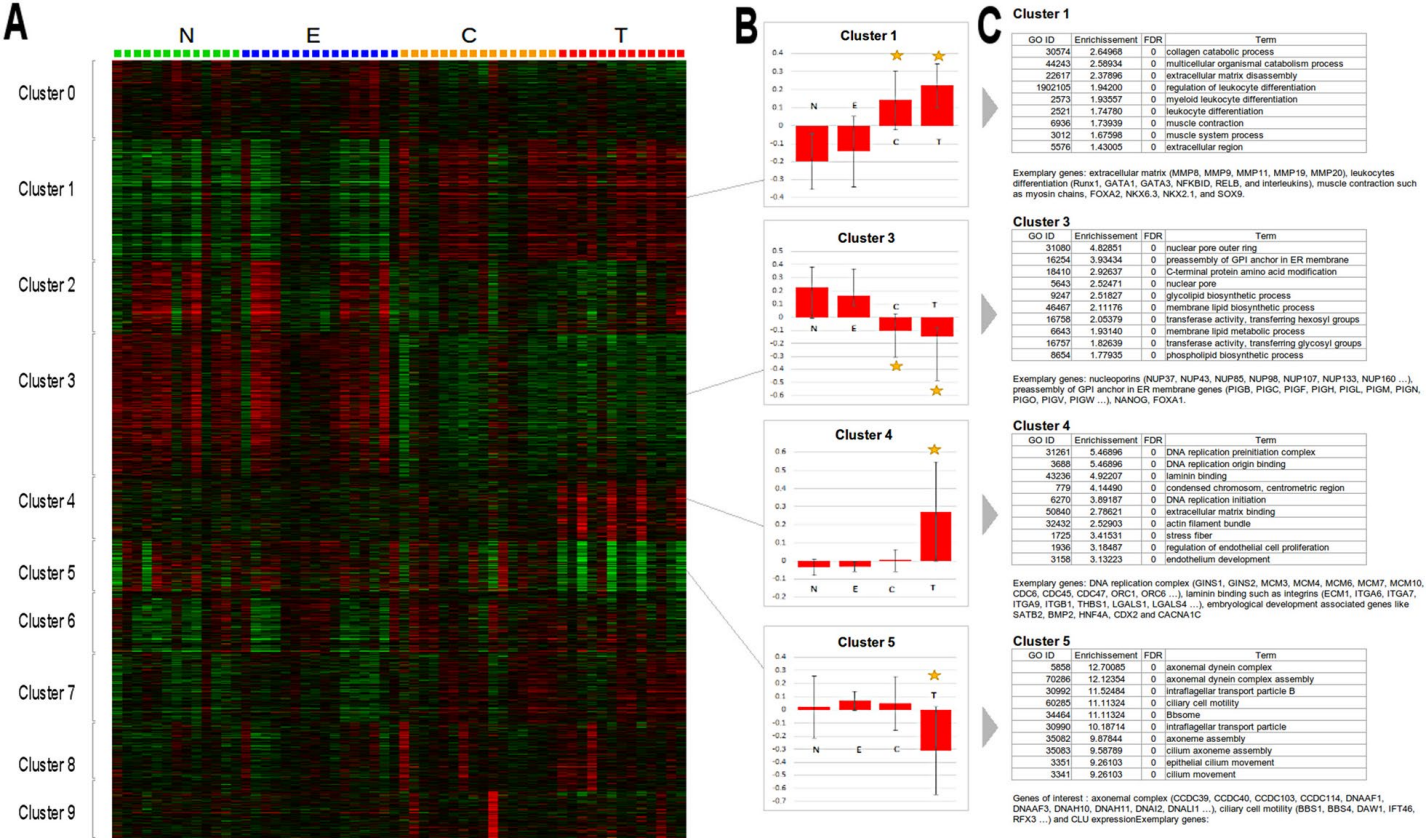
K-means clustering of transcriptome data. (**A**) K-means was used to define 10 groups of genes with similar profiles (*k* = 10, 100 rounds, correlation distance). (**B**) Boxes represent mean profiles of a given cluster. Yellow star means profile significantly different from normal samples (N). Significance was calculated with a student test between the group tested and the normal group, with a Bonferroni correction for multitesting. N: Normal, E: Exposed, C: Contralateral, T: tumor. (**C**) Top-10 annotations of significant clusters and exemplary genes. Clusters were subjected to functional annotations of Gene Ontology using GOminer. GO_ID refers to Gene Ontology unique identifier and Term refers to Term name. Molecular Function, Cellular Component and Biological Process wee pooled together. Enrichment refer to Observed/Expected frequency of GO term. FDR is the corrected *p*-value calculated by bootstrap. A FDR of 0 means inferior to 0.0001

A global transcriptome analysis was performed to find out if it was possible to distinguish the different samples profiles. All results are presented in Fig. 2 as a *k*-means. We identified 4 differential clusters between T and N samples. No differential cluster could be identified between N and E samples. Finally, two clusters could identify healthy subjects samples (E and N) and samples of woodworkers with a tumor (T) even in contralateral sides samples (C). These differences persisted even when adjusting for potential confounders (age or wood dust exposure duration and/or latency).

We then search for functional annotation enrichment of these clusters (Fig. 2).

Cluster 1, overexpressed in C and T, is enriched in genes involved in the extracellular matrix such as collagens, matrix metalloproteinases (*MMP8, MMP9, MMP11, MMP19 and MMP20*), leukocytes differentiation (*Runx1, GATA1, GATA3, NFKBID, RELB* and interleukins) and muscle contraction such as myosin chains. This suggests a change in cellularity (increase in contractile cells and leukocytes) of CT samples. This cluster also includes *FOXA2, NKX6.3, NKX2.1* and *SOX9*.

Cluster 3, underexpressed in C and T samples, is enriched in nuclear pore genes such as nucleoporins (*NUP37, NUP43, NUP85, NUP98, NUP107, NUP133, NUP160*, etc.) and preassembly of GPI anchor in ER membrane genes such as phosphatidylinositol glycan anchor genes (*PIGB, PIGC, PIGF, PIGH, PIGL, PIGM, PIGN, PIGO, PIGV, PIGW*, etc.). This suggests a decrease in protein transport in CT samples. This cluster also contains *NANOG* and *FOXA1*.

Cluster 4, overexpressed in T samples, is enriched in genes involved in DNA replication such as DNA replication complex (*GINS1, GINS2, MCM3, MCM4, MCM6, MCM7, MCM10, CDC6, CDC45, CDC47, ORC1, ORC6*, etc.), and in laminin binding such as integrins (*ECM1, ITGA6, ITGA7, ITGA9, ITGB1, THBS1, LGALS1, LGALS4* …). These genes are involved in cell proliferation and cancers. In particular, ECM1 is implicated in breast cancer, thyroid cancer, hepatocellular carcinoma and other cancers and also in ulcerative colitis. Galectin-1 (*LGALS1*) may act as an autocrine negative growth factor that regulates cell proliferation and is involved in Hodgkin lymphoma. *LGALS4* (Galectin-4) is a protein which exacerbates intestinal inflammation has been previously pointed out as an important gene in ITACs [15]. Cluster 4 contains embryological development-associated genes like *SATB2*, *BMP2 or HNF4A.* Finally, cluster 4 also contains two exemplary genes: *CDX2* involved in ITACs and the calcium channel, voltage-dependent, L type, alpha 1C subunit (*CACNAC*). This suggests an increase in cell division in T samples.

Cluster 5 underexpressed in T samples is strongly enriched in genes involved in cilium functions: axonemal complex (*CCDC39, CCDC40, CCDC103, CCDC114, DNAAF1, DNAAF3, DNAH10, DNAH11, DNAI2, DNALI1*, etc.) and ciliary cell motility (*BBS1, BBS4, DAW1, IFT46, RFX3*, etc.). In this cluster, *CLU* expression was also decreased. Diminution of cluster 5 gene expression in tumors probably reflects the change in cellularity: diminution of ciliary cells due to invasion of tumor epithelial cells.

### ITAC predictor

Hierarchical classification of genes and samples showed a clear separation between N/E and C/T samples. A C/T mean profile was created, and Pearson’s correlation with this profile was calculated for each sample. This predictor was highly significant and had a sensibility of 97% and a specificity of 93% (Additional file 4: Figure 1).

### DNA methylome analysis

**Fig. 3:**
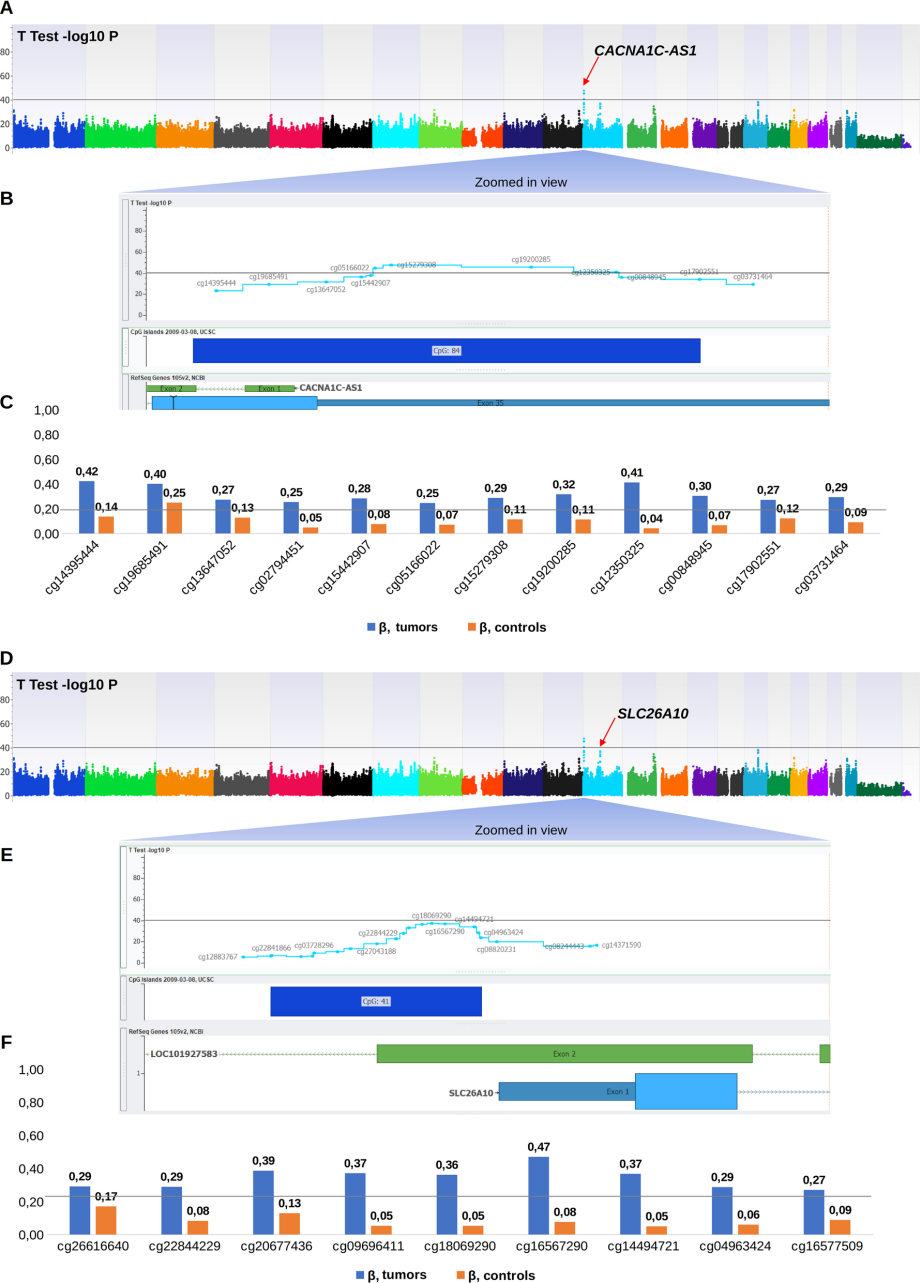
Analyses of Methylome data. (**A**) Epi-Manhattan plot reporting the epigenome-wide association study and the top first significant locus on *CACNA1C/CACNA1C-AS1*. (**B**) Zoomed view on the genomic context of the *CACNA1C/CACNA1C-AS1* locus. (**C**) *β* values of the CpG probes on the *CACNA1C/CACNA1C-AS1* locus in cancerous and non-cancerous nasal cavity samples. The horizontal line corresponding to a *β* value of 0.2 delimits the threshold between unmethylated status and hemimethylated status for each CpG probe. (**D**) Epi-Manhattan plot reporting the epigenome-wide association study and the top first significant locus on *SLC26A10*. (**E**) Zoomed view on the genomic context of the *SLC26A10* locus. (**F**) *β* values of the CpG probes on the *SLC26A10* locus in cancerous and non-cancerous nasal cavity samples. The horizontal line corresponding to a *β* value of 0.2 delimits the threshold between unmethylated status and hemimethylated status for each CpG probe

**Fig. 4:**
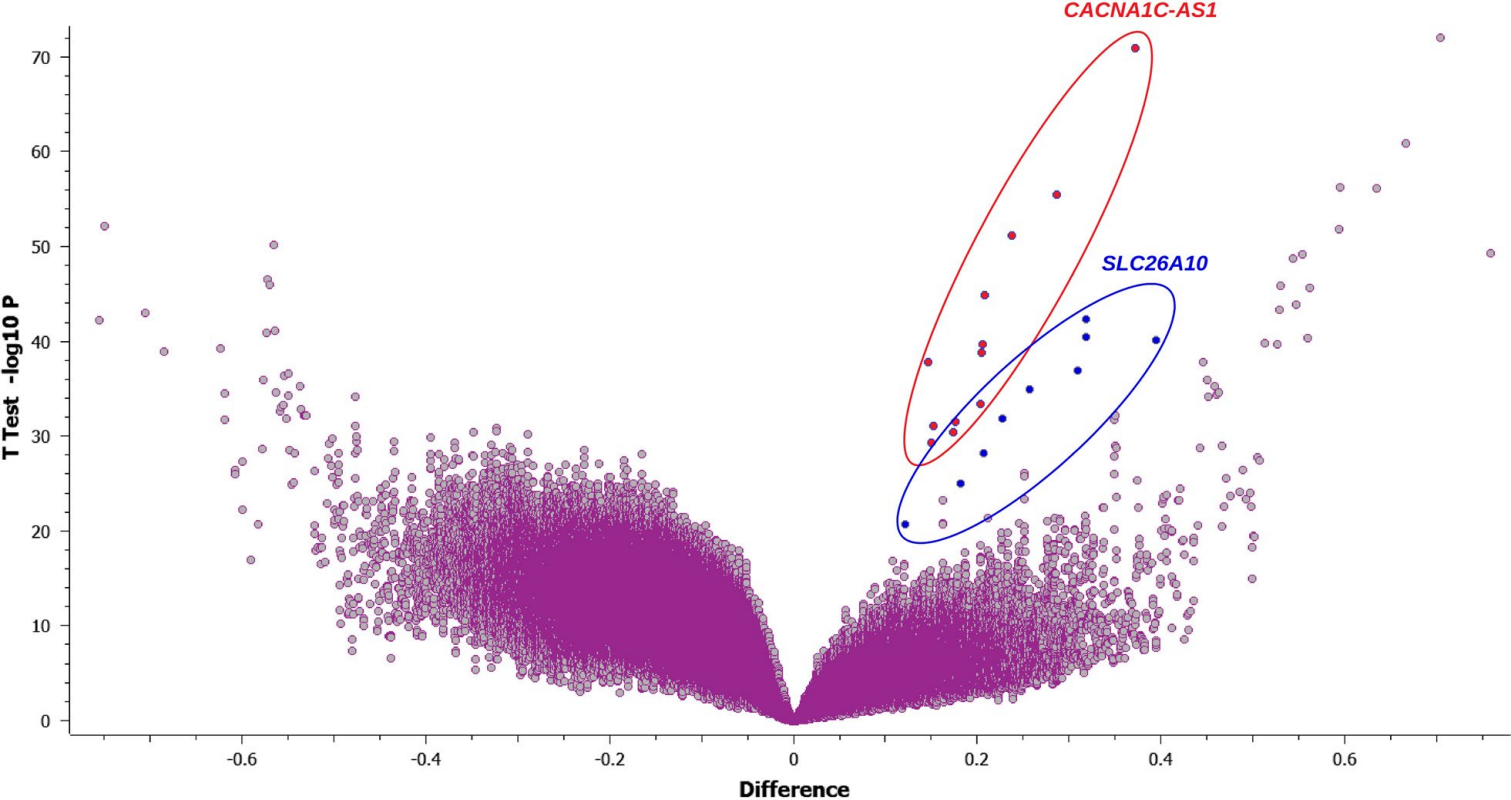
Volcano plot showing the relationship between the magnitude of the difference in *β* values between cancerous and non-cancerous nasal cavity samples and the *P*-values of the *t*-test in the epigenome-wide association study

Of the sixteen tumor samples, eleven could be analyzed for their DNA methylation profile using the Infinium Methylation EPIC array. Among them, four samples exhibited high-quality metrics and were included in the EWAS phase of the study. The remaining samples (*n* = 7) exhibited suboptimal quality metrics and were included in the secondary locus-specific analysis of the study which focused on the top loci retrieved in the EWAS phase. In the EWAS, we retrieved two top loci, namely: *CACNA1C/CACNA1C-AS1* and *SLC26A10*. The first top locus corresponded to the CpG island ‘CpG:84’ which locates in the promoter 5’ region of the *CACNA1C* Antisense RNA 1 (*CACNA1C-AS1*) and the 3’UTR region of the *CACNA1C* gene*.* The CpG probes reported in this locus exhibited a hemimethylated profile (*β* values between 0.2 and 0.6) among tumor samples and a fully unmethylated profile among non-tumor samples (Fig. 3A–C and Table 2). The second top locus retrieved in the EWAS corresponded to the CpG island ‘CpG:41’ which locates in the promoter region of the *SLC26A10* gene. This locus encompasses nine CpG probes that exhibited a hemimethylated profile among tumor samples and a fully unmethylated profile among non-tumor samples (Fig. 3D–F and Table 3). In the secondary locus-specific analysis, the assessment of methylation profiles of the seven tumor samples with a suboptimal quality metrics confirmed the overall hemimethylated profile of the two top loci *CACNA1C/CACNA1C-AS1* and *SLC26A10* (Additional file 1: Tables I and Additional file 2: Table II). The visual inspection of the volcano plot confirmed the clustering of the CpG probes belonging to the *CACNA1C/CACNA1C-AS1* and *SLC26A10* loci (Fig. 4). The DNA methylation profiles of tumor and non-tumor cell lines did not differ significantly regarding the *CDX2* locus.

**Table 2:**
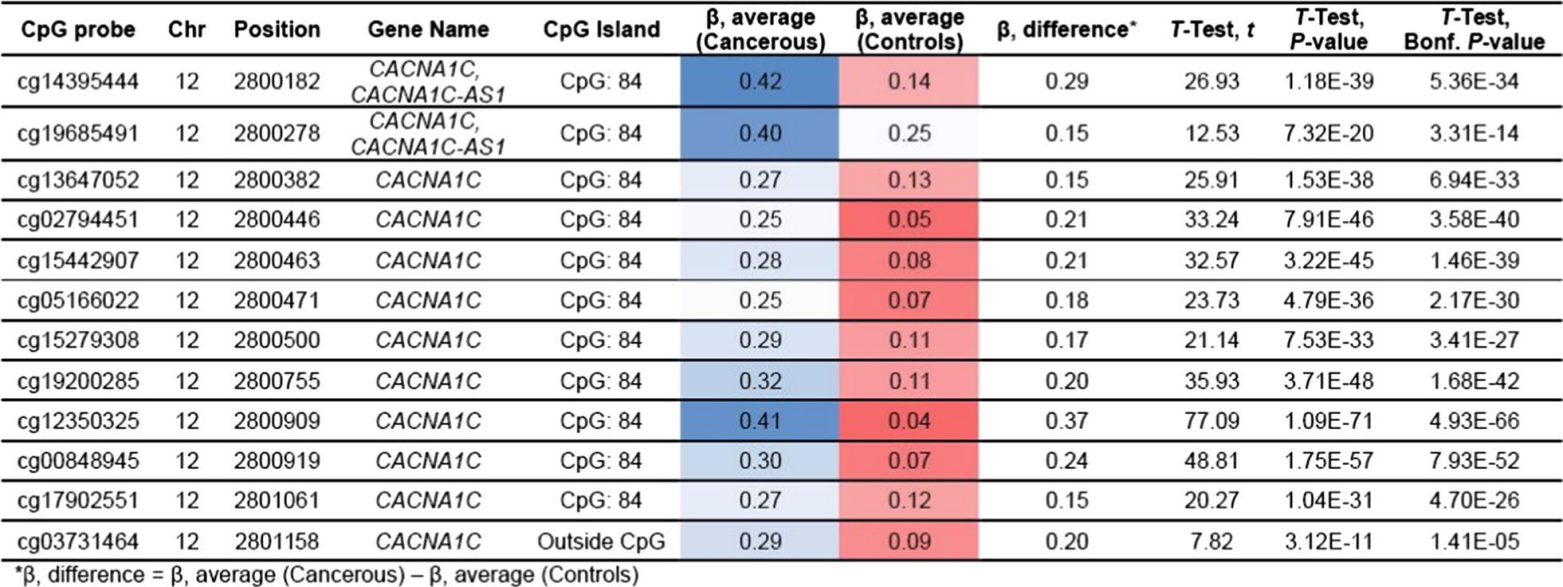
Methylation profiles among cancerous and non-cancerous samples of the CpG probes in the *CACNA1C/CACNA1C-AS1* locus in the epigenome-wide association study

**Table 3:**
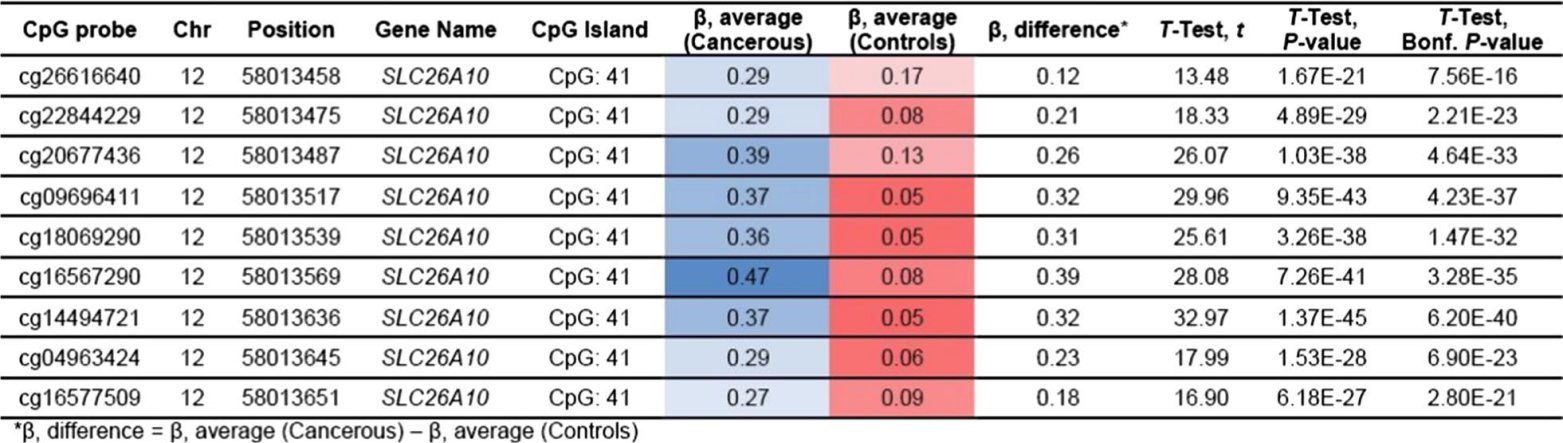
Methylation profiles among cancerous and non-cancerous samples of the CpG probes in the *SLC26A10* locus in the epigenome-wide association study

### Refined microarray data analysis

We looked for expression of *CDX2* and differentially methylated genes: *CACNA1C-AS* and *SLC26A10*. *CACNA1C-AS* and *SLC26A10* expressions were weakly modified, but a significant effect could be observed on *CACNA1C* with a fold change of 1.52. *CDX2* expression was significantly increased in T samples (Fold change of 2.46 for T/N samples), but also in some C samples (Additional file 5: Figure 2).

### Immunochemistry

A strong nuclear staining for *CDX2* was confirmed in all tumor samples (Additional file 6: Figure 3A). *CACNA1C* immunostaining was focally positive in tumor samples while negative in adjacent non-tumor mucosa (Additional file 6: Figure 3B). *SLC26A10* was strongly positive in both tissues (Additional file 6: Figure 3C).

### Replication of our results with dataset GSE17433

We searched for another dataset of sinonasal adenocarcinomas [25] in the Gene Expression Omnibus database (https://www.ncbi.nlm.nih.gov/geo/). After normalization of each dataset, common genes of both studies were selected and sorted according to our clustering. Genes overexpressed in tumors in our study (cluster 4) are also overexpressed in GSE17433 (Additional file 7: Figure 4). Genes underexpressed in tumors in our study (cluster 5) are also underexpressed in GSE17433. We investigated the expression of the Top-100 discriminant genes of our study in GSE17433, and the Top-100 discriminant genes of Tripodi’s study (GSE17433) in our results. Most of these results were concordant (112/143 genes had the same variations, 52/55 when analyzing only genes with significant variations in both studies) (Additional file 3: Table III).

## Discussion

Using a noninvasive well-accepted sampling technique, we have identified several transcription modifications useful for distinguishing different categories of samples, particularly tumor samples. As the predictor allowed to distinguish N/E samples from C/T samples, this study raises the possibility of an easy and noninvasive diagnostic process to detect ITACs and/or high-risk profiles.

When compared to healthy mucosa cells, the main changes in tumor cells transcriptome were in agreement with prior studies [15], carcinogenesis and tumor phenotype: The decreased expression in the ‘cilium’ cluster and the increased expression in the ‘DNA replication initiation’ cluster were consistent with the loss of the respiratory phenotype (with ciliary cells and sero-mucous glands) to the benefit of an intestinal phenotype [1]. There were no significant changes in samples of woodworkers without ITAC: Wood dust exposure is supposed to induce chronic inflammation, but this had no visible impact on the transcriptome. In contrast, woodworkers with ITAC exhibited a different transcriptomic profile both in tumor and in contralateral epithelium: Two clusters were indeed highly differential. The first one mainly consisted of overexpression in collagen and extracellular matrix catabolism, regulation of leukocytes differentiation and might be the witness of a specific inflammation. The second one consisted of underexpression in membrane and nuclear pore activity. Contralateral transcriptome modifications are hardly explained by the influence of the contralateral tumor, as these changes 1/were not limited to inflammation and 2/concerned situations with a very small contralateral tumor unlikely to affect the other side. The slight differences in exposure latency or in woodworkers’ age do not explain this result either, as transcriptomic profiles were still different when adjusting for confounders. Thus, the bilaterality of changes could be explained by a specific sensitivity to wood dust or a different type of exposure. In light of these bilateral modifications, the rarity of bilateral ITACs might appear surprising, but ITACs cancerogenesis seem to rely on additional events and stochastic transformations.

For the time being, these additional events remain unknown, but our study reveals a gene of interest: *CACNA1C*. The hemimethylation of the CPG codon of *CACNA1C-AS* should result in a diminution of its antisense expression, thus indirectly releasing the brake on *CACNA1C* expression that was confirmed in transcriptome analysis and immunochemistry. *CACNA1C* gene is encoding Cav1.2 (α1) subunit, one of the 4 subunit of the L-Type voltage-gated calcium channel, which consists of 24 transmembrane segments and forms the pore through which calcium enters into the cell. Calcium plays a key role in cell proliferation, activating or inhibiting various intracellular enzymes in numerous compartments including the cytosol, organelles and nucleus. Thus, many cancer features such as cell proliferation, apoptosis, metastatic ability or migration are regulated by intracellular calcium oscillations. *CACNA1C* overexpression has already been described in colorectal or gastric adenocarcinomas [16], with *CACNA1C* appearing in the top 10% of the most augmented genes. These alterations both in methylome and in transcriptome underline the particular role of *CACNA1C*: It might therefore be considered as a master gene of ITACs [17].

As for *SLC26A10,* it is one of the 11 genes of the SLC26 gene family of anion transporters and channels. *SLC26A10* mRNA has already been described as downregulated tenfold in gastric carcinoma cells by knockdown of PTP-1B [18]. But this gene is a pseudogene and no functional studies of putative SLC26A10 polypeptides have been reported. In our case, hemimethylation of *SLC26A10* promoter did not seem to significantly decrease *SLC26A10* expression: Therefore, its role might be reduced.

Consistently with its presumed key role in ITACs intestinal differentiation [1, 2, 11], *CDX2* was overexpressed in all tumor samples. Many genes associated with *CDX2* during embryological development were also overexpressed (*NKX2.1, SOX9, BMP2,* most of *HOX* genes, most of *KLF* genes), and our study highlighted gene expression modifications that could participate in the dedifferentiation/redifferentiation process of the original epithelium (such as *OCT3* and *FOXA2* overexpression) so that the hypothesis of a form of oncogenic reprogramming seems consistent. Interestingly, *CDX2* expression was also increased in the contralateral epithelium, while there was no tumor. Thus, *CDX2* overexpression might be detected before the presence of any macroscopically visible lesion. But *CDX2* overexpression alone seems insufficient for tumorigenesis. The analogy between Barrett’s adenocarcinomas and ITACs is interesting because both mucosae share the same embryologic origin and the same transdifferentiation toward an intestinal phenotype when exposed to a specific carcinogen [1]. In Barrett's adenocarcinoma, ectopic CDX2 expression in mice seemed sufficient to induce a tissular differentiation change and cause metaplasia [19] but insufficient to cause degeneration into cancer [20]. Mechanisms might be similar for ITACs. In Barrett's adenocarcinoma, some authors suggested that variations in *CDX2* promoter methylation induced by chronic inflammation may guide intestinal differentiation [21, 22]. The question remained open for ITACs: For Perrone [23], p14(*ARF*) and p16(*INK4a*) were frequently hypermethylated, while for Perez-Ordonez [24], promoter methylation of *MMR* genes does not play a role in the pathogenesis of ITAC. In our study, *CDX2* expression was *not* associated with methylation changes.

## Conclusion

Thanks to a noninvasive brushing technique, it has been possible to identify transcriptomic and methylation modifications that are consistent with phenotypic profiles and ITACs natural history, thus making it possible to identify subjects with an ITAC. Our study revealed differences that have not yet been described for *CACNA1C-AS* and paves the way for a simple and noninvasive diagnosis method for ITAC in male woodworkers. These results need to be confirmed by larger studies.

## Material and methods

### General design and population

The general design of the study is depicted in Fig. 1. The first set of samples were taken on woodworkers (group 1) with unilateral nasal intestinal adenocarcinomas which were operated on during a three-year period (2014–2016) within the scope of the REFCOR (French Network of Rare Head and Neck Tumors). Samples were collected in olfactory clefts by a noninvasive swabbing technique (for transcriptome analysis), and by biopsies (for DNA methylome and western blot analysis). Then, we included wood dust-exposed volunteers (group 2) and healthy non-exposed volunteers (group 3) as controls from June 2016 to August 2016 and collected samples from the most accessible olfactory cleft (Fig. 1). For ethical reasons, we did not perform biopsies on healthy subjects: A previously published methylation dataset of olfactory clefts of asthmatic patients (without wood dust exposure) was used as controls for methylome analysis [25]. In order to limit biases due to sex, age, genetic abnormalities or inflammatory disease (unrelated to wood dust exposure), the criteria for non-inclusion were as follows for all subjects: 1/age inferior to 50 years 2/previous history of nasal irradiation 3/chronic inflammatory disease affecting the nasal cavity (nasal polyposis, systemic disease, cystic fibrosis), 4/any genetic disease known to be a risk factor for cancer (xeroderma pigmentosum, chromosomal aberrations, abnormalities in DNA repair).

The transcriptome and methylome analyses were conducted in parallel: We first compared T (transcriptome) and T’ (methylome) samples (tumor samples) with N (transcriptome) and N’ (methylome) samples (healthy non-exposed samples). We also investigated wood dust-induced transcriptome modifications of exposed (without tumor) woodworkers’ samples (E) and contralateral, non-tumor sides (C samples) of woodworkers with tumors: As E and N samples exhibited no significant difference, they were grouped for further comparison with C and T samples. The transcriptome analysis enabled to design a predictor based on most differential genes. The results of transcriptome and methylome analyses were then cross-checked to refine the transcriptome analysis. The transcriptome analysis was thereafter replicated on another dataset.

### Swabbing technique and samples collection

Local anesthesia was performed using 1% Xylocaine spray. The brush (bronchial cytologic brush Asept Inmed diameter 1.8 mm/length 1200 mm) was inserted into the nasal cavity, within a curved sucker to guide the introduction into the olfactory cleft. The brush was moved forward in the olfactory cleft, until its tip was in contact with the anterior wall of the sphenoid, then moved back from 2 cm, still sheathed. Then the brush was pushed forward outside of its sheath, and moved 3 times in a back and forth motion of 2 cm length. When brushing was complete, the brush was withdrawn into the sheath and retracted through the curved sucker. The brush was cut and placed in 0.5 mL of RNAlater medium. The samples were sent to the pathology laboratory, in the same conditions than frozen sections, to be immediately vortexed and frozen (tube vertically placed in liquid nitrogen, then stored at − 80 °C). For woodworkers with tumor, biopsies were additionally taken in both olfactory clefts, at the same level, and sent to the pathology laboratory in the same conditions.

All subjects were asked to rate 1/perceived usefulness of the screening method, 2/acceptability, 3/pain. They were also asked if they would accept to be screened again by the same method.

### Purification of RNA

Briefly, samples were incubated with 50 μL of TRIzol (Invitrogen, Carlsbad, CA) reagent at room temperature for 15 min to extract DNA. Two methods were used: with cellular suspension only (after centrifugation) or with cellular suspension and brushes. Samples were vortexed. Phase separation was ensured by adding 200 μL of chloroform. Samples were centrifuged at 10,000*g* for 10 min, and glycogen was added as a carrier. Then, we added 0.5 mL of isopropanol per each milliliter of the clear phase. After mixing and precipitation (10 min), we collected the precipitated RNA by centrifugation at 10,000 g in a centrifuge for 10 min at 4 °C. After decantation of the supernatant, we removed remaining liquid and immediately resuspended the pellet (without drying) in 1X SDS solubilization buffer. We added NaOAc to 3 M, re-extracted the solution with phenol and ensured precipitation with ethanol.

### Transcriptome analysis

RNA quantities were determined using the Nanodrop Spectrophotometer ND-1000 and the Qubit™ 2.0 Fluorometer with the Qubit™ RNA HR Assay Kit. We assessed RNA integrity using RNA 6000 PicoChips with Agilent 2100 BioAnalyzer. All RNA samples had a low RIN number < 6. Transcriptome profiling was conducted using Affymetrix Genechip Human Gene 2.0 ST Array (Thermofisher) following GeneChip WT Pico Reagent Kit Manual Target Preparation for GeneChip® Whole Transcript (WT) Expression Arrays UserGuide P/N 703262 Rev. 2

### Microarray data analyses

Analyses were performed as previously described [17, 26]. Scanned signals were quantified from all microarrays by GenePix Pro software version 5.1 (Axon Instruments, Union City, CA). Signals were normalized against a median profile using the LOWESS method [27]. Genes with similar profiles were grouped using *k*-means (*k* = 10, 100 runs) algorithm [28] on log-transformed and median center genes with Pearson coefficient as distance and average linkage clustering method. The initialization step consisted of randomly distribute the genes into one of 10 clusters and calculate their centroids. Then, the algorithm proceeded by alternating between two steps: an assignment step aiming to redistribute the observations into the clusters, associating each observation with the nearest centroid (Euclidean method), then an update steps aiming to recalculate the new centroids for each cluster. The redistribution of observations and recalculation of centroids was performed according to the same principle until the stability of the distribution or the maximum level (100) was reached. For each sample type (N, E, C, T) and each cluster, mean and standard deviation were calculated (Fig. 2B). Groups were compared with the Standard Student T-test with a Bonferroni correction for multitesting. Data were visualized using JavaTreeView [29]. Gene signatures were functionally annotated with GoMiner [30] and Gene Ontology [31].

The specific microarray data analysis was focused on CDX2 and its main downstream genes and genes identified by DNA methylome analysis (CACNA1C and SLC26A10).

We designed a gene predictor to predict nasal intestinal adenocarcinoma existence in patients, by selecting the best genes separating N/E from C/T samples. To achieve this goal, we selected the Top-100 genes from cluster 1 and the Top-100 genes from cluster 3. We calculated a mean CT profile and calculated Pearson’s correlation with this profile for each sample. Separation quality was estimated by Fisher’s exact test, sensitivity by calculating True positive rate and specificity by calculating true negative rate. Samples giving a weak correlation (between − 0.25 and 0.25) were considered as unpredictable.

### DNA methylome analyses

We carried out bisulfite conversion of 600 ng of tumor tissue DNA using EZ DNA Methylation kit (Zymo Research, Proteigene, Saint-Marcel, France). The genome-wide profiling of DNA methylome was determined using the Infinium Methylation EPIC BeadChip array (Illumina, Paris, France), according to the manufacturer’s instructions. The Infinium Methylation EPIC BeadChip provides coverage of 850,000 CpG probes in enhancer regions, gene bodies, promoters and CpG islands. The arrays were scanned on an Illumina iScan® system, and raw methylation data were extracted using Illumina’s Genome Studio methylation module. For each CpG probe, the methylation level was described as a *β* value, ranging between 0 (fully unmethylated CpG probe) and 1 (fully methylated CpG probe). Background correction and normalization was implemented using the SWAN method (R Package Minfi) [32]. We visually inspected the whole-genome distribution of the CpG probes according to their *β* value. Samples that did not show a methylation profile consistent with a beta distribution were excluded from the epigenome-wide association study (EWAS) and were used in the secondary locus-specific analysis. In the EWAS, we compared the whole DNA methylome profile of nasal cavity tumor samples with that of 72 non-tumor nasal cavity methylomes profiles retrieved from the GEO database (GSE65163) [15]. For each CpG probe, we compared the mean *β* values between tumor and non-tumor samples using *t*-test with Bonferroni correction to account for the multiple testing issue. Due to the low sample size, and considering the exploratory approach of our analysis, we used the smoothed *P*-value transformation by converting nominal *P*-values obtained from the *t*-test to smoothed *P*-values using a window radius of 3, as previously reported [33]. All statistical analyses were performed using the SNP & Variation Suite (v8.8.1; Golden Helix, Inc., Bozeman, MT, USA).

### Immunochemistry

To confirm the observed transcriptomic and methylome modifications, tumor sections were reacted with specific antibodies (*CDX2* (clone DAK-CDX2, 1:100 dilution, DAKO, Carpinteria, CA, USA), *CACNA1C* (HPA039796, 1:50 dilution, Sigma-Aldrich, St Louis, MO, USA), *SLC26A10* (primary antibody: HPA044719, 1:200 dilution, Sigma-Aldrich, St Louis, MO, USA), then were stained immunohistochemically by the avidinbiotin complex method. tumor tissue stainings were compared to non-tumor adjacent mucosa stainings as controls.

### Data availability

The datasets supporting the conclusions of this article will be available in the Gene Expression Omnibus repository.

## Supplementary Information

**Additional file 1: Table I.** Methylation profiles of the CpG probes among tumor samples in the secondary locus-specific analysis of *CACNA1C/CACNA1C-AS1*.

**Additional file 2: Table II.** Methylation profiles of the CpG probes among tumor samples in the secondary locus-specific analysis of *SLC26A10*

**Additional file 3: Table III.** Replication of our results on GSE17433. We investigated the expression of the Top-100 discriminant genes of our study in GSE17433 and the expression of the Top-100 discriminant genes of GSE17433 in our results and compared both. On 143 common genes, 112 had the same variations, 52/55 when analyzing only genes with significant variations in both studies. Among important genes, CACNA1C, SLC26A10, CDX2 or SATB2 expressions were not available in GSE17433

**Additional file 4: Figure 1.** Exemplary genes expression. *CDX2* and the three differentially methylated genes (*CACNA1C*, *CACNA1C–AS1*, *SLC26A10*) mean expression in the N/E/C/T groups. Yellow star means profile significantly different from Normal samples with correction for multitesting. Fold change (FC_T/N_) is calculated from unlogged original data, P is the p-value of the N to T comparison

**Additional file 5: Figure 2.** Immunohistochemistry validation. (**A**) ***CDX2***, tumor tissue. Original magnification × 10. Tumor cells exhibited strong nuclear positivity for CDX2. (**B**) ***CACNA1C***, tumor tissue. Tumor cells exhibited focal cytoplasmic positivity for *CACNA1C*. Original magnification × 20. (**C**) ***SLC26A10***, tumor tissue. Tumor cells exhibited strong and constant membrane positivity for *SLC26A10*. Original magnification × 40

**Additional file 6: Figure 3.** Results of the predictor built from the most differential genes

**Additional file 7: Figure 4.** Replication of our results with dataset GSE17433. The figure depicts the K-means clustering of our study and GSE17433. Lines correspond to genes and columns correspond to samples. Each cell represents the level of expression of one gene in one sample (lower expression is figured in green, higher expression in red). N = normal samples, E = samples of exposed individuals, without tumor, C = contralateral samples, T = tumor samples. Genes of cluster 4 are overexpressed in tumor samples of both studies and genes of cluster 5 are underexpressed in tumor samples of both studies

## Data Availability

The datasets used and/or analyzed during the current study are available from the corresponding author on request. Data will be available in the Gene Expression Omnibus repository in a few weeks (AO and RH) but have not been submitted at the time of submission of this manuscript.

## Abbreviation

ITAC: Nasal intestinal-type adenocarcinomas

## References

[1] De Gabory L, Conso F, Barry B, Stoll D (2009). Carcinogenesis of the ethmoidal adenocarcinoma due to wood dust. Rev Laryngol Otol Rhinol (Bord).

[2] Gallet P, Nguyen DT, Russel A, Jankowski R, Vigouroux C, Rumeau C (2018). Intestinal and non-intestinal nasal cavity adenocarcinoma: Impact of wood dust exposure. Eur Ann Otorhinolaryngol Head Neck Dis.

[3] Demers PA, Kogevinas M, Boffetta P, Leclerc A, Luce D, Gérin M (1995). Wood dust and sino-nasal cancer: pooled reanalysis of twelve case-control studies. Am J Ind Med.

[4] Carton M, Goldberg M, Luce D (2002). [Occupational exposure to wood dust. Health effects and exposure limit values]. Rev Epidemiol Sante Publique.

[5] Choussy O, Ferron C, Védrine P-O, Toussaint B, Liétin B, Marandas P (2008). Adenocarcinoma of ethmoid: a GETTEC retrospective multicenter study of 418 cases. Laryngoscope.

[6] Jankowski R, Georgel T, Vignaud JM, Hemmaoui B, Toussaint B, Graff P (2007). Endoscopic surgery reveals that woodworkers’ adenocarcinomas originate in the olfactory cleft. Rhinology.

[7] Georgel T, Jankowski R, Henrot P, Baumann C, Kacha S, Grignon B (2009). CT assessment of woodworkers’ nasal adenocarcinomas confirms the origin in the olfactory cleft. AJNR Am J Neuroradiol.

[8] Bonato M, Piantanida R, Riva C, Cis C, Capella C (1989). Intestinal-type adenocarcinoma of the nose and paranasal sinuses. Histological and immunohistochemical study of 14 cases. Acta Otorhinolaryngol Ital.

[9] Choi HR, Sturgis EM, Rashid A, DeMonte F, Luna MA, Batsakis JG (2003). Sinonasal adenocarcinoma: Evidence for histogenetic divergence of the enteric and nonenteric phenotypes. Hum Pathol.

[10] Donhuijsen K, Hattenberger S, Schroeder HG (2004). [Nasal sinus carcinoma after wood dust exposure. Morphological spectrum of 100 cases]. Pathologe.

[11] Kennedy MT, Jordan RC, Berean KW, Perez-Ordoñez B (2004). Expression pattern of CK7, CK20, CDX-2, and villin in intestinal-type sinonasal adenocarcinoma. J Clin Pathol.

[12] Vivanco B, Llorente JL, Perez-Escuredo J, Alvarez Marcos C, Fresno MF, Hermsen MA (2011). Benign lesions in mucosa adjacent to intestinal-type sinonasal adenocarcinoma. Patholog Res Int.

[13] Wilhelmsson B, Lundh B, Drettner B, Stenkvist B (1985). Effects of wood dust exposure and diethylnitrosamine. A pilot study in Syrian golden hamsters. Acta Otolaryngol.

[14] Porez F, de Pommerol MJ, Krief P, Conso F, Stoll D, de Gabory L (2011). Assessment of nasal fibroscopy to explore olfactory cleft. Otolaryngol Head Neck Surg.

[15] Tripodi D, Quéméner S, Renaudin K, Ferron C, Malard O, Guisle-Marsollier I (2009). Gene expression profiling in sinonasal adenocarcinoma. BMC Med Genomics.

[16] Wang CY, Lai MD, Phan NN, Sun Z, Lin YC (2015). Meta-analysis of public microarray datasets reveals voltage-gated calcium gene signatures in clinical cancer patients. PLoS ONE.

[17] Chen G, Broséus J, Hergalant S, Donnart A, Chevalier C, Bolaños-Jiménez F (2015). Identification of master genes involved in liver key functions through transcriptomics and epigenomics of methyl donor deficiency in rat: relevance to nonalcoholic liver disease. Mol Nutr Food Res.

[18] Alper SL, Sharma AK (2013). The SLC26 gene family of anion transporters and channels. Mol Aspects Med.

[19] Barros R, Freund JN, David L, Almeida R (2012). Gastric intestinal metaplasia revisited: function and regulation of CDX2. Trends Mol Med.

[20] Balbinot C, Armant O, Elarouci N, Marisa L, Martin E, De Clara E (2018). The Cdx2 homeobox gene suppresses intestinal tumorigenesis through non-cell-autonomous mechanisms. J Exp Med.

[21] Wu W, Bhagat TD, Yang X, Song JH, Cheng Y, Agarwal R (2013). Hypomethylation of noncoding DNA regions and overexpression of the long noncoding RNA, AFAP1-AS1, in Barrett's esophagus and esophageal adenocarcinoma. Gastroenterology.

[22] Guo M, House MG, Suzuki H, Ye Y, Brock MV, Lu F (2007). Epigenetic silencing of CDX2 is a feature of squamous esophageal cancer. Int J Cancer.

[23] Perrone F, Oggionni M, Birindelli S, Suardi S, Tabano S, Romano R (2003). TP53, p14ARF, p16INK4a and H-ras gene molecular analysis in intestinal-type adenocarcinoma of the nasal cavity and paranasal sinuses. Int J Cancer.

[24] Perez-Ordonez B, Huynh NN, Berean KW, Jordan RC (2004). Expression of mismatch repair proteins, beta catenin, and E cadherin in intestinal-type sinonasal adenocarcinoma. J Clin Pathol.

[25] Yang IV, Pedersen BS, Liu AH, O’Connor GT, Pillai D, Kattan M (2017). The nasal methylome and childhood atopic asthma. J Allergy Clin Immunol.

[26] Baron D, Dubois E, Bihouee A, Teusan R, Steenman M, Jourdon P (2011). Metaanalysis of muscle transcriptome data using the MAD Muscle database reveals biologically relevant gene patterns. BMC Genomics.

[27] Yang YH, Dudoit S, Luu P, Lin DM, Peng V, Ngai J (2002). Normalization for cDNA microarray data: a robust composite method addressing single and multiple slide systematic variation. Nucleic Acids Res.

[28] De Hoon MJ, Imoto S, Nolan J, Miyano S (2004). Open source clustering software. Bioinformatics.

[29] Saldanha AJ (2004). Java Treeview—extensible visualization of microarray data. Bioinformatics.

[30] Zeeberg BR, Feng W, Wang G, Wang MD, Fojo AT, Sunshine M (2003). GoMiner: a resource for biological interpretation of genomic and proteomic data. Genome Biol.

[31] Ashburner M, Ball CA, Blake JA, Botstein D, Butler H, Cherry JM (2000). Gene ontology: tool for the unification of biology. The Gene Ontology Consortium. Nat Genet.

[32] Aryee MJ, Jaffe AE, Corrada-Bravo H, Ladd-Acosta C, Feinberg AP, Hansen KD (2014). Minfi: a flexible and comprehensive bioconductor package for the analysis of Infinium DNA methylation microarrays. Bioinformatics.

[33] Gueant JL, Chery C, Oussalah A, Nadaf J, Coelho D, Josse T (2018). APRDX1 mutant allele causes a MMACHC secondary epimutation in cblC patients. Nat Commun.

