# Supplementary figures and images for: Integrative genomics analysis of nasal intestinal-type adenocarcinomas demonstrates the major role of CACNA1C and paves the way for a simple diagnostic tool in male woodworkers
Source: Clin Epigenetics. 2021 Sep 25;13:179. doi: 10.1186/s13148-021-01122-5 (PMC8467244; doi:10.1186/s13148-021-01122-5)

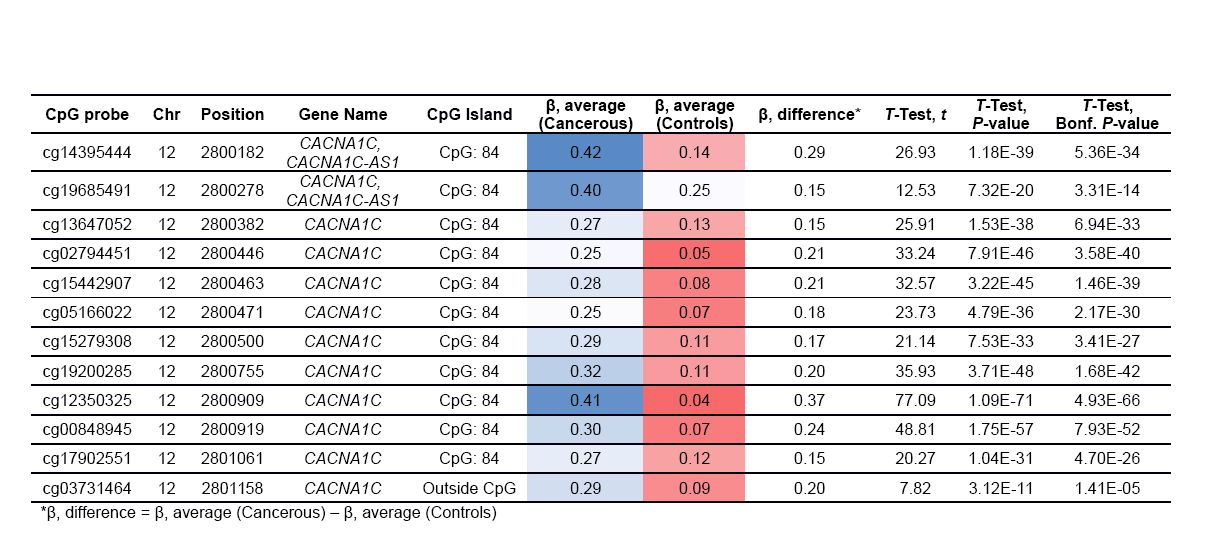

Supplement: Supplementary file 1 — Additional file 1: Table I. Methylation profiles of the CpG probes among tumor samples in the secondary locus-specific analysis of CACNA1C/CACNA1C-AS1. [file 13148_2021_1122_MOESM1_ESM.jpg]

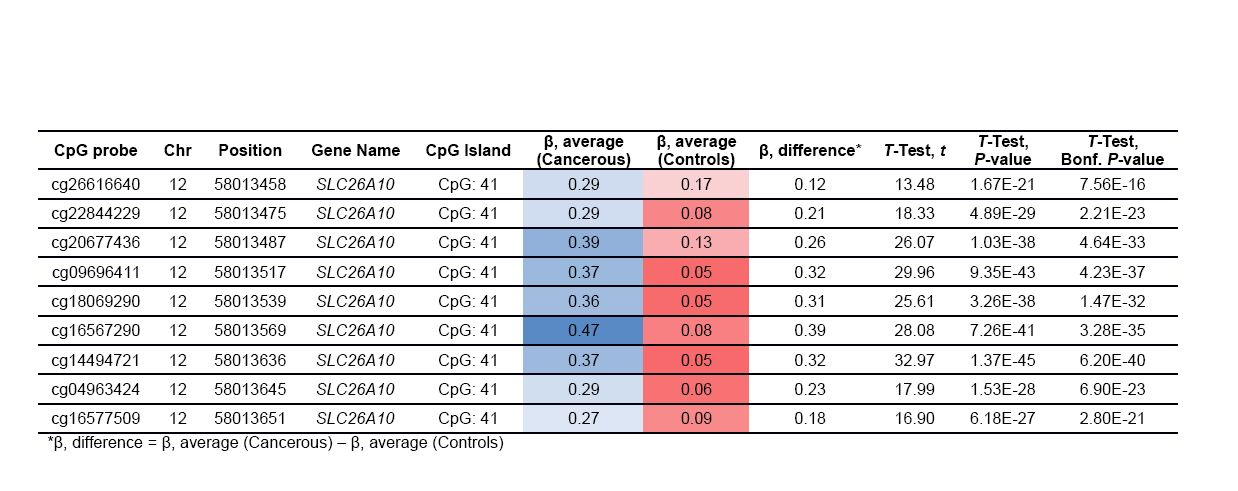

Supplement: Supplementary file 2 — Additional file 2: Table II. Methylation profiles of the CpG probes among tumor samples in the secondary locus-specific analysis of SLC26A10 [file 13148_2021_1122_MOESM2_ESM.jpg]

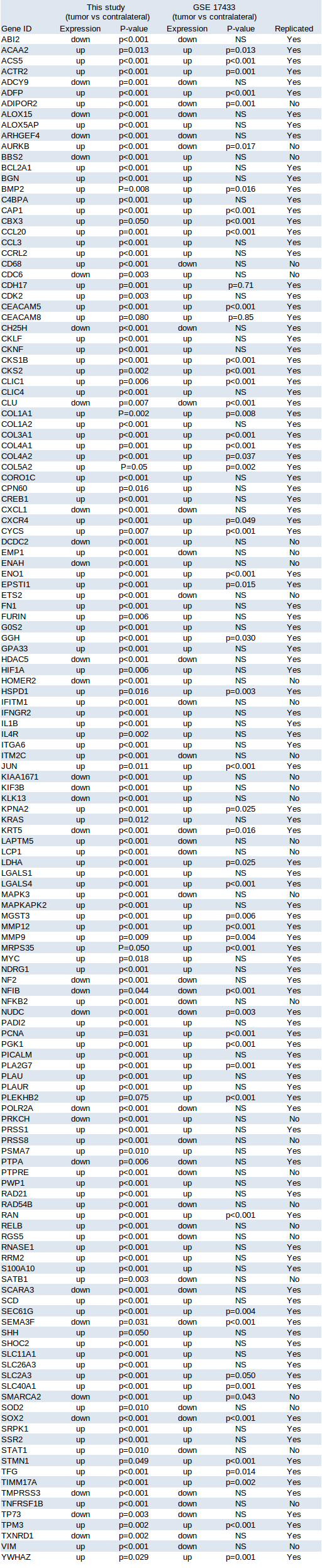

Supplement: Supplementary file 3 — Additional file 3: Table III. Replication of our results on GSE17433. We investigated the expression of the Top-100 discriminant genes of our study in GSE17433 and the expression of the Top-100 discriminant genes of GSE17433 in our results and compared both. On 143 common genes, 112 had the same variations, 52/55 when analyzing only genes with significant variations in both studies. Among important genes, CACNA1C, SLC26A10, CDX2 or SATB2 expressions were not available in GSE17433 [file 13148_2021_1122_MOESM3_ESM.png]

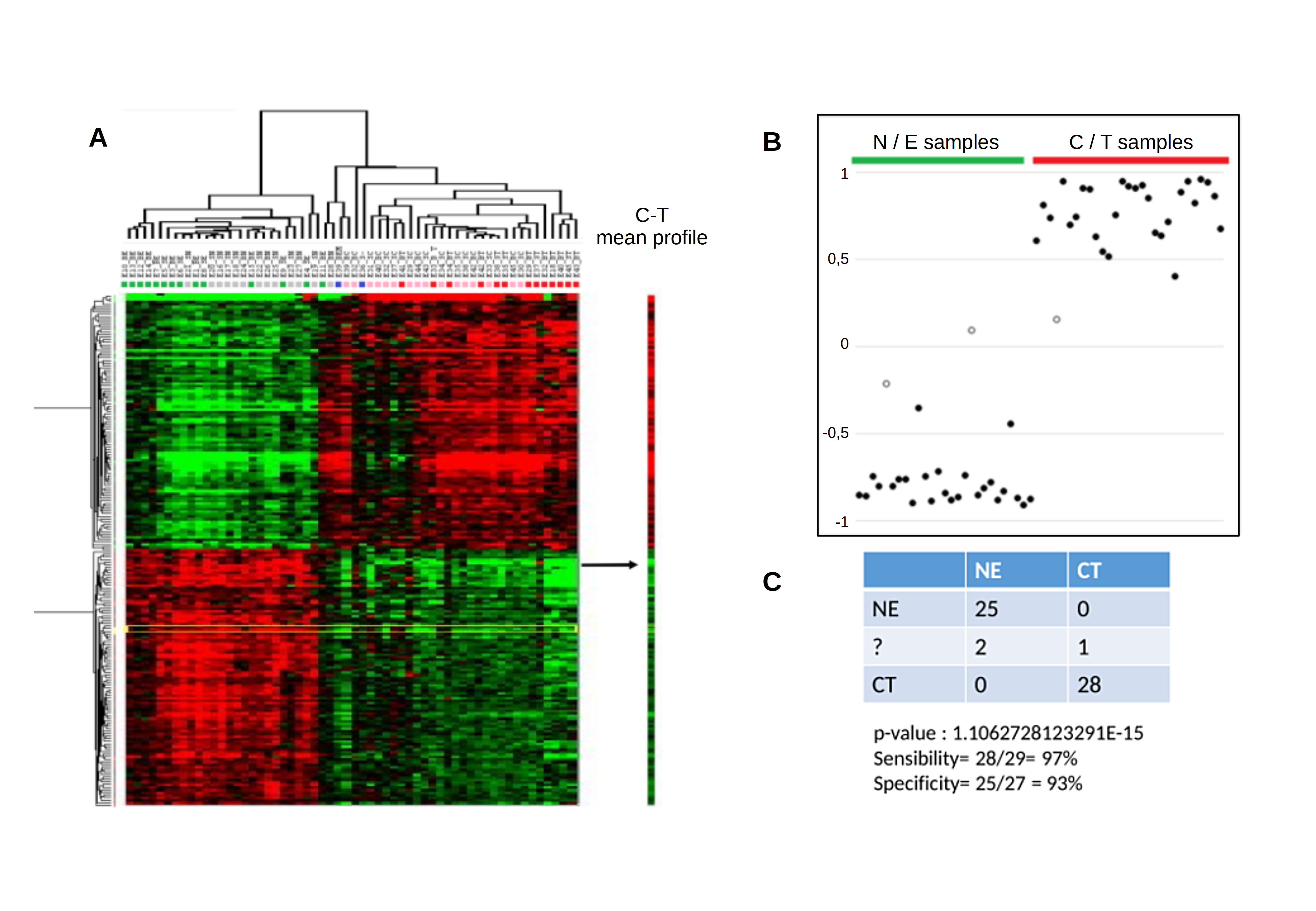

Supplement: Supplementary file 4 — Additional file 4: Figure 1. Exemplary genes expression. CDX2 and the three differentially methylated genes (CACNA1C, CACNA1C–AS1, SLC26A10) mean expression in the N/E/C/T groups. Yellow star means profile significantly different from Normal samples with correction for multitesting. Fold change (FCT/N) is calculated from unlogged original data, P is the p-value of the N to T comparison [file 13148_2021_1122_MOESM4_ESM.tif]

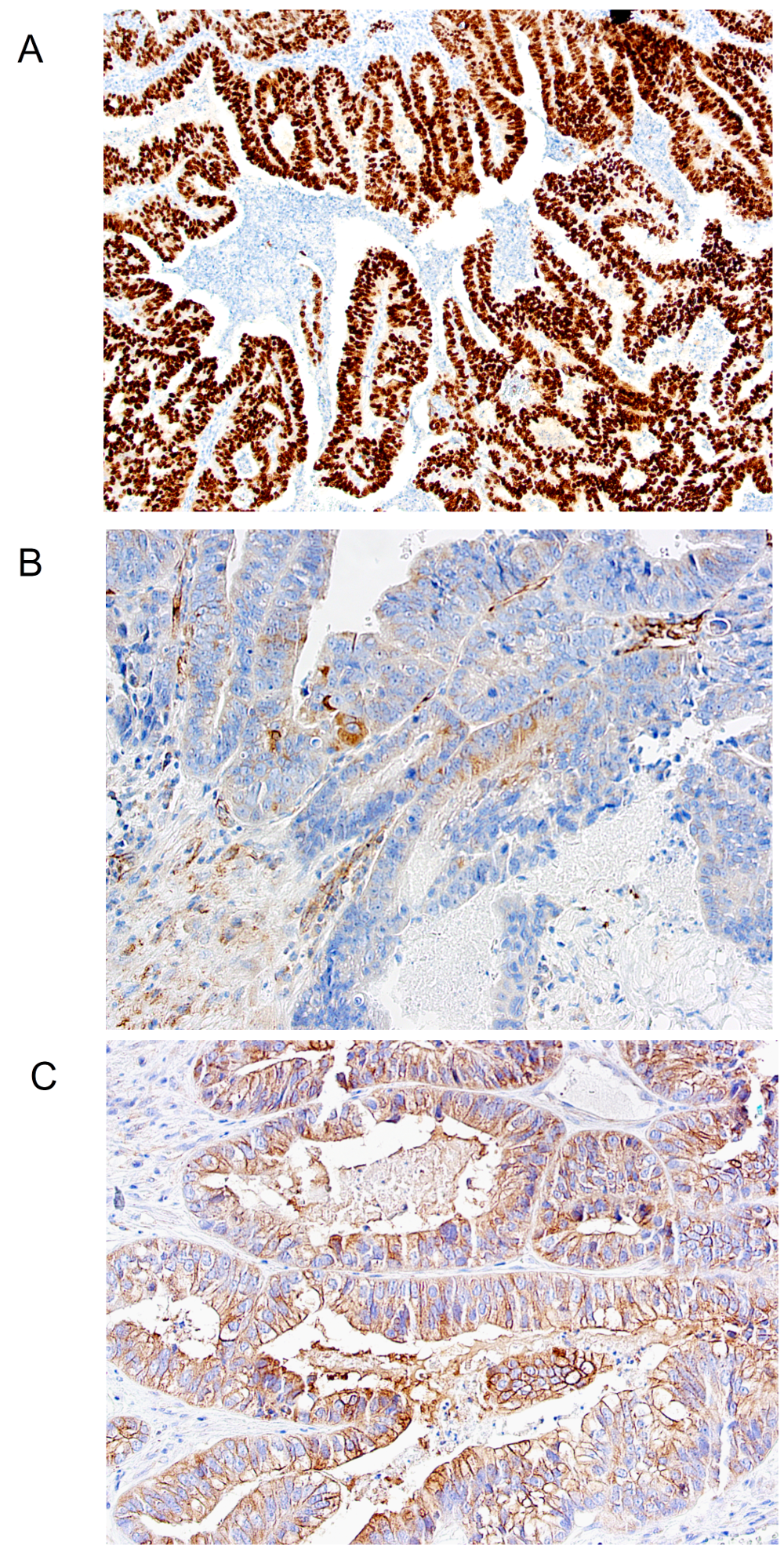

Supplement: Supplementary file 6 — Additional file 6: Figure 3. Results of the predictor built from the most differential genes [file 13148_2021_1122_MOESM6_ESM.png]

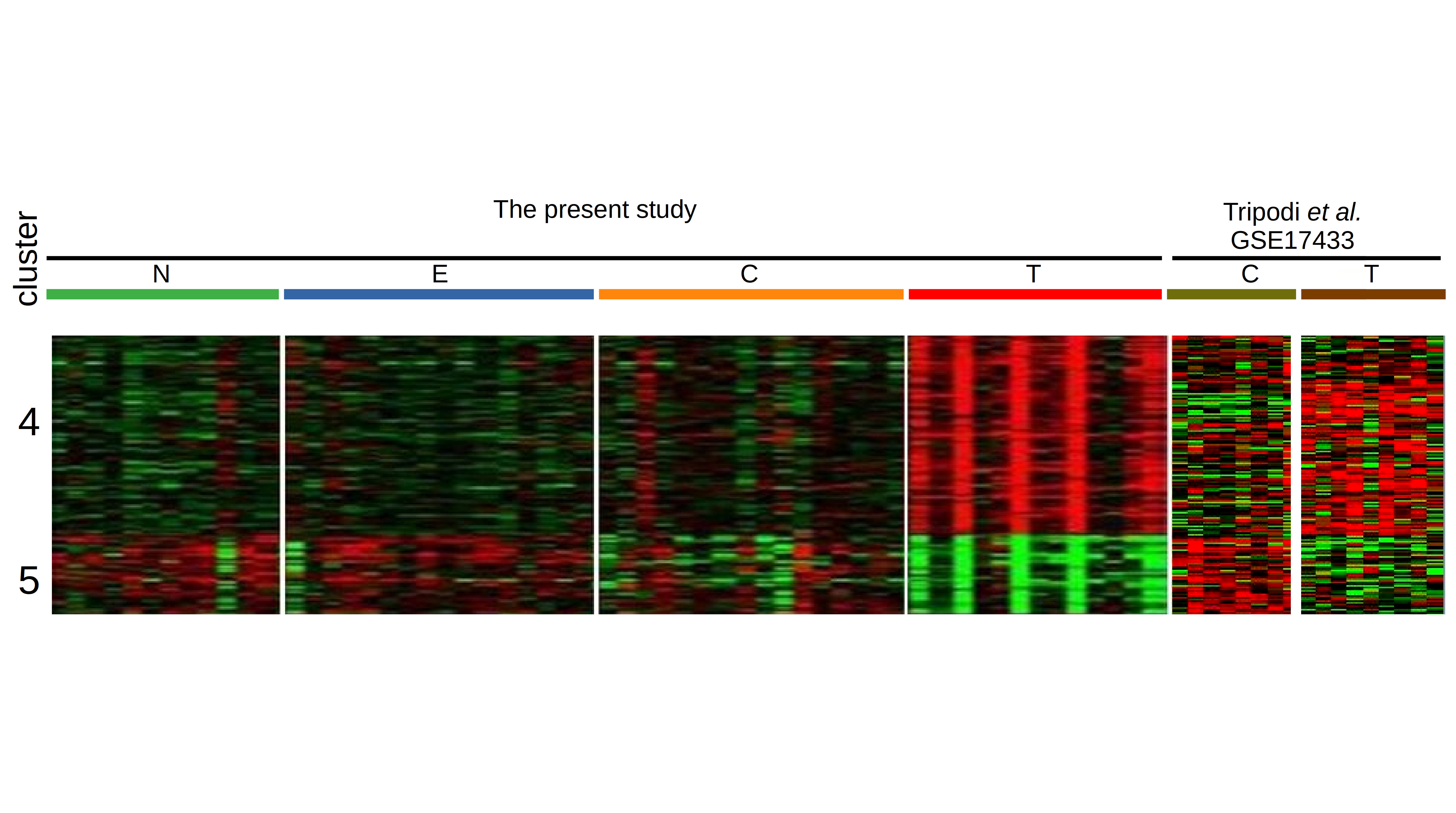

Supplement: Supplementary file 7 — Additional file 7: Figure 4. Replication of our results with dataset GSE17433. The figure depicts the K-means clustering of our study and GSE17433. Lines correspond to genes and columns correspond to samples. Each cell represents the level of expression of one gene in one sample (lower expression is figured in green, higher expression in red). N = normal samples, E = samples of exposed individuals, without tumor, C = contralateral samples, T = tumor samples. Genes of cluster 4 are overexpressed in tumor samples of both studies and genes of cluster 5 are underexpressed in tumor samples of both studies [file 13148_2021_1122_MOESM7_ESM.tif]
